# Exposure contrasts associated with a liquefied petroleum gas (LPG) intervention at potential field sites for the multi-country household air pollution intervention network (HAPIN) trial in India: results from pilot phase activities in rural Tamil Nadu

**DOI:** 10.1186/s12889-020-09865-1

**Published:** 2020-11-26

**Authors:** Sankar Sambandam, Krishnendu Mukhopadhyay, Saritha Sendhil, Wenlu Ye, Ajay Pillarisetti, Gurusamy Thangavel, Durairaj Natesan, Rengaraj Ramasamy, Amudha Natarajan, Vigneswari Aravindalochanan, A. Vinayagamoorthi, S. Sivavadivel, R. Uma Maheswari, Lingeswari Balakrishnan, S. Gayatri, Srinivasan Nargunanathan, Sathish Madhavan, Naveen Puttaswamy, Sarada S. Garg, Ashlinn Quinn, Josh Rosenthal, Michael Johnson, Jiawen Liao, Kyle Steenland, Ricardo Piedhrahita, Jennifer Peel, William Checkley, Thomas Clasen, Kalpana Balakrishnan

**Affiliations:** 1 Department of Environmental Health Engineering, ICMR Center for Advanced Research on Air Quality, Climate and Health, Faculty of Public Health, Sri Ramachandra Institute of Higher Education and Research (Deemed University), Porur, Chennai, 600116 India; 2 Gangarosa Department of Environmental Health, Rollins School of Public Health, Emory University, Atlanta, GA USA; 3 Division of Epidemiology and Population Studies, Fogarty International Center, National Institutes of Health, Bethesda, MD USA; 4 Berkeley Air Monitoring Group, Berkeley, California USA; 5 Department of Environmental and Radiological Health Sciences, Colorado State University, Fort Collins, CO USA; 6 Division of Pulmonary and Critical Care, School of Medicine, Johns Hopkins University, Baltimore, MD USA

**Keywords:** HAPIN trial, LPG intervention, Household air pollution, PM_2.5_, Personal exposures, Pregnant women, India

## Abstract

**Background:**

The Household Air Pollution Intervention Network (HAPIN) trial aims to assess health benefits of a liquefied petroleum gas (LPG) cookfuel and stove intervention among women and children across four low- and middle-income countries (LMICs). We measured exposure contrasts for women, achievable under alternative conditions of biomass or LPG cookfuel use, at potential HAPIN field sites in India, to aid in site selection for the main trial.

**Methods:**

We recruited participants from potential field sites within Villupuram and Nagapattinam districts in Tamil Nadu, India, that were identified during a feasibility assessment. We performed.

(i) cross-sectional measurements on women (*N* = 79) using either biomass or LPG as their primary cookfuel and (ii) before-and-after measurements on pregnant women (*N* = 41), once at baseline while using biomass fuel and twice – at 1 and 2 months – after installation of an LPG stove and free fuel intervention. We involved participants to co-design clothing and instrument stands for personal and area sampling. We measured 24 or 48-h personal exposures and kitchen and ambient concentrations of fine particulate matter (PM2.5) using gravimetric samplers.

**Results:**

In the cross-sectional analysis, median (interquartile range, IQR) kitchen PM2.5 concentrations in biomass and LPG using homes were 134 μg/m3 [IQR:71–258] and 27 μg/m3 [IQR:20–47], while corresponding personal exposures were 75 μg/m3 [IQR:55–104] and 36 μg/m3 [IQR:26–46], respectively. In before-and-after analysis, median 48-h personal exposures for pregnant women were 72 μg/m3 [IQR:49–127] at baseline and 25 μg/m3 [IQR:18–35] after the LPG intervention, with a sustained reduction of 93% in mean kitchen PM2.5 concentrations and 78% in mean personal PM2.5 exposures over the 2 month intervention period. Median ambient concentrations were 23 μg/m3 [IQR:19–27). Participant feedback was critical in designing clothing and instrument stands that ensured high compliance.

**Conclusions:**

An LPG stove and fuel intervention in the candidate HAPIN trial field sites in India was deemed suitable for achieving health-relevant exposure reductions. Ambient concentrations indicated limited contributions from other sources. Study results provide critical inputs for the HAPIN trial site selection in India, while also contributing new information on HAP exposures in relation to LPG interventions and among pregnant women in LMICs.

**Trial registration:**

ClinicalTrials.Gov. NCT02944682; Prospectively registered on October 17, 2016.

**Supplementary Information:**

The online version contains supplementary material available at 10.1186/s12889-020-09865-1.

## Introduction

Household air pollution (HAP) from the use of solid fuels (such as wood, animal dung, and crop residue) for cooking and heating is a leading risk factor for population health [1]. The health burden, largely borne by the rural poor of low and middle income countries (LMICs), includes a wide range of impacts on respiratory, cardiovascular, and pregnancy related outcomes [2]. In India, biomass fuels are the primary household energy source for approximately 846 million people, more than 55% of the nation’s population [3, 4]. In 2017, 1.24 million deaths, or 12.5% of all deaths in India, were attributable to air pollution and 0.48 million were attributable specifically to HAP [3].

HAP exposure and its potential adverse health effects have been examined in India over the last several decades [5]. Recent intervention studies in India [6–13] report improvements in lung function indicators [7, 8, 11], lower systolic and diastolic blood pressure [6, 9, 12], and less self-reported respiratory symptoms associated with the use of cleaner biomass cookstoves and clean fuels such as biogas, electricity, and Liquefied Petroleum Gas (LPG). In most instances, however, the observed health improvements have been marginal and inconsistent, potentially on account of stove stacking (where cleaner and traditional stoves are used together), other sources of personal exposures (including ambient exposures), and the inability [7, 8, 11] of interventions to achieve/sustain health relevant exposure reductions [14–16].

Globally, several field-based randomized controlled trials (RCTs) have been conducted to evaluate the effect of clean cooking interventions on multiple health outcomes (including child pneumonia [17, 18], birth weight [19], adult lung function [20, 21], and blood pressure [22]). Some of the earliest efforts included cleaner biomass cookstove studies in rural Mexico and the RESPIRE (Randomized Exposure Study of Pollution Indoors and Respiratory Effects) trial in Guatemala [17]. More recent RCTs have either used cleaner biomass cook-stoves (such as in Malawi [18, 23, 24], Honduras [25], India [18], and Nepal [26]); clean fuel/stoves (such as an ethanol stove in Nigeria [19, 22], LPG in Peru [27], or a combination of cleaner biomass cookstoves and clean fuels such as in Ghana [28]). Similar to observational studies described above, the results from RCTs reported thus far, too, fail to provide a consistent picture of health benefits. Lower-than-expected exposure reductions associated with interventions [17], moderate intervention adherence [18], and cross-contamination from other sources [18, 19] are thought be responsible for the statistically insignificant health improvements in the intervention group when compared to the control group [19, 22]. There is currently no data from clinical trials for exclusive use of LPG stoves and fuel in India, the country with the largest population relying on biomass solid fuels and with some of the largest Government-led initiatives underway to increase access to LPG [29, 30].

The Household Air Pollution Intervention Network (HAPIN) Trial has been designed to overcome many of the evidence gaps for health benefits from HAP interventions, while also attempting to address the challenges encountered in previous HAP RCTs. It aims to assess the health benefits (i.e. increased birth weight, reduced pneumonia incidence and improved growth [length-for-age/stunting] among children up to 1 year of age, and lower blood pressure among older women) of a LPG cook-stove and fuel intervention prospectively in four Intervention Research Centers (IRCs): Guatemala, India, Peru and Rwanda (https://clinicaltrials.gov/ct2/show/NCT02944682, [31–33]).

One of the key inputs for successful conduct of HAP RCTs is the availability of detailed site-specific information gathered prior to the main trial through formative research. After an initial scoping phase to identify potential sites based on feasibility (Thangavel et al., *in preparation)*, we undertook an assessment of expected exposure contrasts associated with the planned intervention, potentially avoiding costly investments on sites that could fail either due to lack of intervention feasibility or lack of exposure reduction.

The RCTs described above have seldom reported detailed results on site selection. The notable exception to this is the RESPIRE trial in Guatemala that reported results from multiple pilot studies in the 1990s prior to the main trial conducted between 2002 and 2004 [34, 35]. This included establishing the local availability and acceptability of the intervention (i.e. the “plancha” improved cookstove), establishing the potential for exposure reduction, and validating measurements of exposure for carbon monoxide. Most other HAP RCTs provide relatively limited information on potential for exposure contrast at putative trial sites, instead using considerations of high HAP levels at baseline, local logistics, stove or fuel distribution feasibility, and institutional support as the primary reasons for site selection [15, 24, 36].

A central aim of the HAPIN trial is to maximize external validity by undertaking research in diverse yet representative populations and settings. At the same time, HAPIN is an efficacy trial designed to determine the health effects that might be achieved with reductions in exposure to HAP. We report exposure measurement results from the pilot phase of the HAPIN trial in India with the principal objective of establishing exposure contrasts at the proposed Indian HAPIN field sites. A secondary objective was to contribute to the refinement of planned exposure assessment protocols for the overall HAPIN trial. Although we focused on PM_2.5_ exposures on women and pregnant women in this pilot exercise, the HAPIN main trial will additionally measure carbon monoxide and black carbon and include exposures for children.

Specifics of site selection in India, including feasibility-based assessments for how sites were chosen, are being reported elsewhere (Thangavel et al., *in preparation*). Trial-wide exploration of potential exposure contrasts for all sites is reported in Liao and Kirby (*under review*).

## Methods

### Initial site selection

**Fig. 1:**
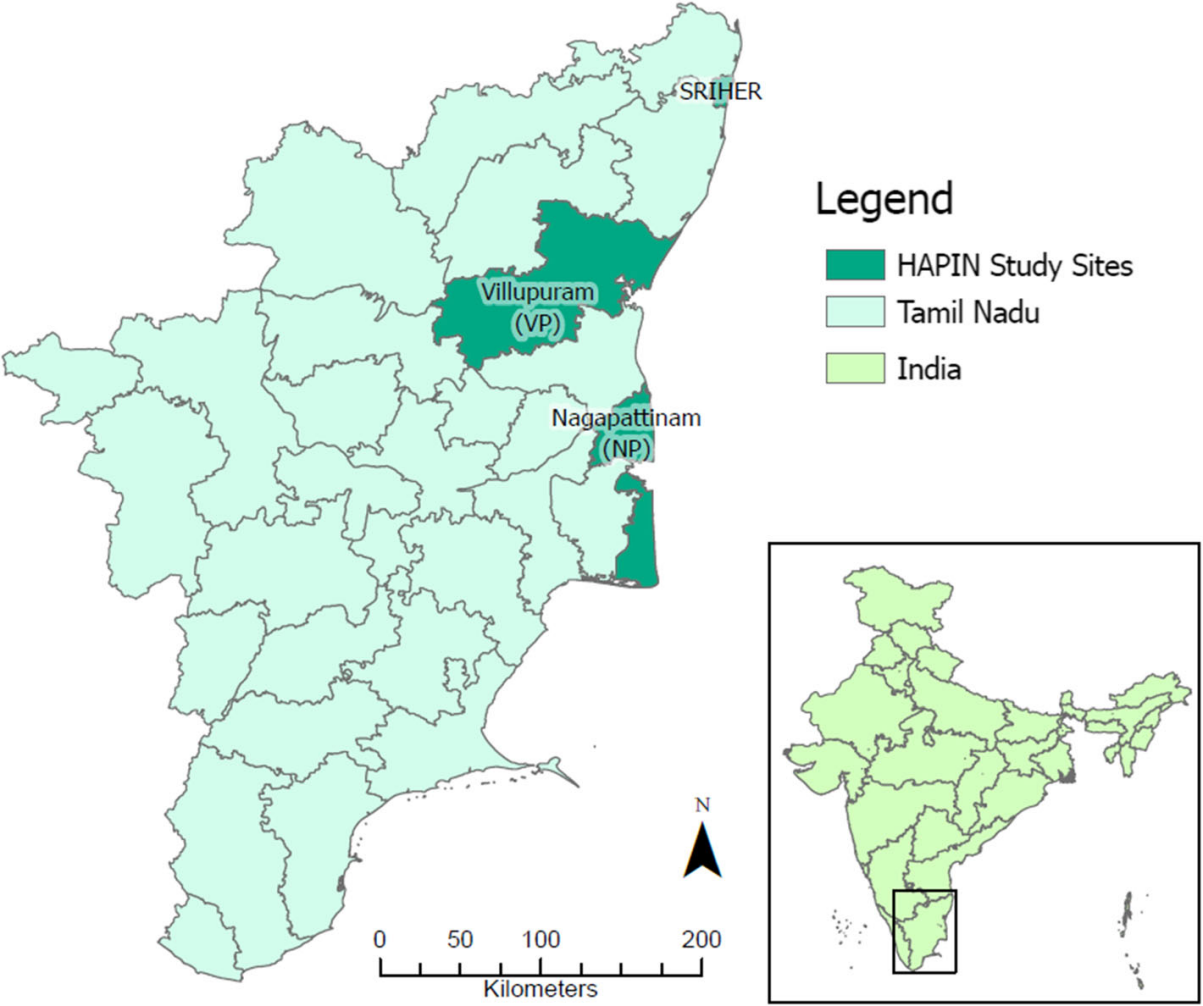
Candidate study districts (Villupuram-VP and Nagapattinam-NP) for the HAPIN Trial in India (Map not to scale. VP and NP sites are at a distance of ~ 250 km and 500 km respectively from the host institution, Sri Ramachandra Institute for Higher Education and Research (SRIHER). (Source: https://www.freeworldmaps.net/asia/india/tamilnadu/where-is-tamilnadu.jpg; https://commons.wikimedia.org/wiki/File:India_Tamil_Nadu_Nagapattinam_district.svg; adapted from under a Creative Commons Attribution-Share Alike 3.0 Unported license [more information at https://creativecommons.org/licenses/by-sa/3.0/deed.en]. Adaptations including changing of color of highlighted areas and creation of single panel)

Site selection and piloting activities in India were conducted between November 2016 and December 2017 in accordance with a detailed Formative Research Protocol. This included a scoping phase to assess feasibility, performed between November 2016 and March 2017 to identify potential HAPIN field sites, followed by assessment of exposure contrast at the candidate sites between April 2017 and December 2017. We used multiple criteria to select candidate sites during the scoping phase (Thangavel et al., *in preparation*). Briefly, these include low rates of LPG penetration, low anticipated uptake of LPG among primary biomass cook-fuel users over the 5 years of the trial, and low anticipated air pollution cross-contamination from neighbouring households and other ambient sources (such as waste incineration sites, brick kilns, crop burning etc.). This exercise identified five blocks (an administrative revenue unit defined for implementation of welfare schemes) in the districts of Villupuram and Nagapattinam in the southern state of Tamil Nadu, India, as potential candidate sites (Fig. 1). The study block (Kalrayan Hills) at Villupuram (VP) district is located at an altitude of 800 m above sea level, while the study blocks of Keezhvelur, Kezhayur, Thalainayiru, and Vedaranyam in Nagapattinam (NP), a coastal district, are at an average elevation ranging from 10 to 50 m above sea level.

### Study design

We designed an exposure monitoring strategy at candidate study blocks in VP and NP districts to establish the potential exposure contrasts that could be expected with an LPG intervention. The protocol in India broadly followed common pilot phase protocols designed for implementation across HAPIN countries with some differences across sites necessitated by local access to air pollution monitors, housing types, economic and cultural conditions (Liao and Kirby, *under review*).

The exposure monitoring strategy was two-pronged with (i) cross-sectional measurements on women, under routine conditions of either primary biomass or LPG cook-fuel use and (ii) before-after measurements on pregnant women over a 3 month period: once at baseline while using biomass fuel, and at 1 and 2 months after installing an LPG stove and providing free fuel. We measured 24 or 48 h personal exposures and kitchen, near-household (outdoor), and ambient concentrations of fine particulate matter (PM_2.5_) using gravimetric samplers (described below). We also elicited participant feedback to design acceptable clothing for wearing the monitors.

### Inclusion criteria

For cross-sectional measurements, we recruited women (*N* = 79) aged (18–64) with a BMI < 40 kg/m^2^ from a convenience sample of households selected from each of the 5 identified blocks across the two districts, with representation from households that used biomass (*N* = 48) or LPG(*N* = 31) as the primary cookfuel. Women were approached directly by study staff to ascertain eligibility and were administered informed consent. For these measurements, we recruited women over two successive time periods. In the first period we recruited women aged 18–35 years, followed by women aged 35–64 years in the second period. The age groups were chosen to mirror eligibility for enrollment as pregnant women or older adult women in the main trial.

For before-and-after measurements, we recruited pregnant women (*N* = 41) aged 18–35 years who used biomass exclusively as a cooking fuel, with a gestational age of 9 to < 20 weeks (the same gestational age window for recruitment into the main HAPIN trial) by screening those seeking routine antenatal care at local primary health centers in each of the five study blocks. Eligibility was based on: (i) ultrasound assessments at registered ultrasound centers in the study area to determine gestational age; (ii) reported exclusive biomass use and the absence of an LPG connection at baseline, so as to pilot the process of securing an LPG connection as required in the main trial; and (iii) reported availability of the participant over the 3 month study period at the same residence (without an intention to move). Smokers and women with pre-existing health conditions (including previously diagnosed hypertension) were excluded for cross-sectional as well as before-after measurements. Also, since these measurements were performed sequentially, there was no instance of more than one eligible woman in the same household.

The inclusion criteria across the two arms were designed such that we could: (i) test the feasibility of being able to perform exposure measurements on pregnant and other older women, similar to those expected to be enrolled in the main trial; and (ii) evaluate exposure contrasts under conditions of routine LPG use versus HAPIN efficacy trial conditions (i.e. providing a free LPG stove and fuel).

### Intervention package

An LPG intervention was provided to pregnant women enrolled for before-and-after measurements. For each household with an eligible pregnant woman, the study team prepared the required documentation for securing a licensed LPG connection (access to LPG in India requires refills to be supplied via authorized distributors of public sector oil marketing companies). Upon receipt of the connection license, which includes an LPG regulator, the study team supplied a two-burner stainless steel LPG stove and a hose connecting the stove to the regulator, which is attached to an LPG cylinder (Figure S1). Installation of the LPG cylinder and instructions for safe use were provided by the authorized distributor. LPG cylinder refills were provided at no cost to households by the distributor for 3 months after installation. Cylinders and stoves were left with the participant at the end of the study period.

All households received a simple behavioural reinforcement message [37] developed by the Behavioural and Economics Core (BEC) of the HAPIN trial. As part of that message, we placed a poster on the intervention stove, demonstrating the feasibility of cooking typical dishes using available household utensils, without modifying cooking practices.

### Intervention adherence

Field staff visited households every week to monitor adherence and confirm exclusive LPG use. This included recording any visible signs of biomass stove use at the time of visit (such as presence of cinder/ash on the stove or a warm stove); asking participants if they had to use the biomass stove for any specific purpose (over the preceding week); eliciting feedback on the ease of routine cooking with LPG, including from available family members; and recording primary and/or secondary biomass stove dismantling/disablement. Queries regarding LPG stove repairs were also addressed during the weekly visits.

### Air pollution measurements

We conducted 24- or 48-h measurements of PM_2.5_ concentrations in the kitchen and wherever possible, in the near-home outdoor space, with simultaneous personal exposure measurements on women. Cross-sectional assessments included 24 or 48-h measures and were performed over two separate 8 week periods to include young women (aged 18–35) and older women (aged 35–64) respectively. Before-after measurements were performed on pregnant women (aged 18–35) at baseline, and again at 1 and 2 months after the LPG intervention, and included only 48-h measures.

Details of PM_2.5_ measurements and filter weighing protocols are furnished in Supplementary Information (SI). Briefly, gravimetric personal and micro-environmental area samples were collected using (i) three different types of constant flow pumps – the Casella TuffPro (Casella Measurement, Bedford, UK), Airchek XR5000, and Universal PCXR8 pumps (SKC, Eighty Four, PA, USA) connected to a Triplex personal cyclone (BGI, Cambridge, MA, USA); using (ii) the Ultrasonic Personal Air Sampler (UPAS, Colorado State University, Fort Collins CO, USA) with built-in cyclone [38]; and using (iii) the Enhanced Children’s MicroPEM (ECM, RTI International, Durham, NC, USA) with built-in impactor. Wherever feasible, we co-located these monitors to assess inter-monitor correlation in a subset of households (SKC/Casella samplers with UPAS (*n* = 41) or ECM samplers (*n* = 21)). We also changed filters over two consecutive 24-h periods on the SKC/Casella samplers (*n* = 15) to compare 24 vs. 48 h averages in the same household. The ECM samplers also collected real-time nephelometric data that are not included in analyses presented in this paper. 48-h ambient measurements were performed using the MiniVol™ (Airmetrics, Oregon, USA) at the VP and NP sites by placing the sampler (one in each site) on the rooftop of a willing community resident’s home.

We collected 21 field blank filters (7 for 15 mm filters used in ECM samplers, 16 for 37 mm filters used in the SKC/Casella/UPAS samplers and 2 for 47 mm filters used in the MiniVol samplers) to account for changes in filter mass due to handling and transportation. Final PM_2.5_ mass depositions were blank adjusted. The median value of field blanks was 2 μg for 15 mm filters, 2 μg for 37 mm filters, and 3 μg for 47 mm filters.

We excluded a total of 53 measurements with (a) sampling times that deviated from the expected sample duration (*n* = 44, 7.7%) by ±20% or (b) all filters with damage, such as holes or tears (*n* = 9, 1.6%). Our final dataset included 521 gravimetric PM_2._5 samples from the following environments: 216 (48 h, *n* = 142 and 24 h, *n* = 74) from the kitchen area, 95(48 h, *n* = 64 and 24 h, *n* = 31) from the near outdoor environment and 210 (48 h, *n* = 143 and 24 h, *n* = 67) personal exposures.

We conducted household surveys using REDCap (Vanderbilt University, Nashville, TN, USA) to assess cooking practices, identify other potential sources of air pollution, and record self-reported compliance in wearing the monitoring equipment. Customised vests (referred to here as “wearables”) and equipment housings (such as stands) were tested in other pre-existing SRIHER field sites prior to deployment at newly identified HAPIN sites. We also elicited detailed qualitative participant feedback on the acceptability of wearables, devices, and device housings (such as stands) with respect to comfort of fabric choice and vest designs, weight of alternative monitoring devices, and inconvenience caused by equipment housings/stands.

## Results

A total of 120 households were enrolled from 17 villages across the two sites between April 2017 and December 2017. In the cross-sectional arm, 96 women were screened and 79 were enrolled, with 6 unwilling to participate and 11 found ineligible (BMI > 40 kg/m^2^ or history of high blood pressure). In the before-and-after arm, 81 were screened and 41 were enrolled, with 3 women unwilling to receive an ultrasound, 11 unwilling to participate, and 26 found ineligible (LPG usage at baseline (17); gestational age < 9 weeks or > 20 weeks (9)).

### Participant and household characteristics

Table 1 summarizes relevant household and participant characteristics.

**Table 1:** Characteristics of study participants and households enrolled for exposure measurements

	Cross-sectional measurements	Before-and-after measurements
**Characteristics of participants**		
No of women	79	41
Age in years, mean (SD)	37.4 (10.4)	23.6 (2.8)
Number of school years, mean (SD)	2.5 (3.3)	7.5 (5.1)
Primary cook, n(%)	71 (88)	39 (96)
**Household characteristics**		
**Kitchen Characteristics**		
Fully enclosed (roof with 4 walls)n(%)	80 (99)	33 (80)
Kitchen size in m^2^, mean (SD)	9.6 (4.0)	10.1 (4.0)
Kitchen height in m, mean (SD)	3.0 (0.7)	2.8 (0.6)
**Kitchen type,n(%)**		
Separate building	16 (20)	7 (18)
Separate room attached to main house	45 (56)	19 (46)
Main living area in house	20 (25)	10 (24)
**Primary cooking fuel/stove,n(%)**		
Biomass	51 (63)	N/A^a^
LPG stove	30 (37)	N/A
**Number of stoves used,n(%)**		
One Stove	40 (49)	29 (71)
Two stoves	31 (38)	11 (27)
More than two stoves	10 (12)	1 (2)
**Other HAP sources**		
**Main lighting source,n(%)**		
Kerosene lamp	4 (5)	4 (10)
Electricity	77 (95)	37 (90)
**Secondary lighting source,n(%)**		
Kerosene lamp	56 (69)	27 (66)
Other	2 (2)	2 (5)
**Main heating source,n(%)**		
No heating	56 (69)	25 (61)
Cooking fire	25 (31)	16 (39)
**Other Sources, n(%)**		
Garbage Burning	17 (21)	11 (27)
Use of mosquito coils	27 (33)	7 (17)
Smoke from neighbour’s home	30 (37)	12 (29)
Smoker in household^b^	33 (41)	20 (49)
Incense burning	64 (79)	36 (88)

^a^Primary cooking fuel/stove for before-after measurements is not provided as only exclusive biomass users were eligible to be enrolled at baseline

^b^Only non-smoking women were eligible to be enrolled. All reported smokers in households were male

A majority of households cooked in enclosed indoor kitchens with limited ventilation, with a significant proportion (27–38%) reporting use of more than one stove. Most women (88–96%) enrolled were also the primary cooks in the family. Table S1 in Supplementary Information (SI) provides a summary by site, with no major notable differences across sites.

### Feedback on wearables, equipment, and equipment housing

We made multiple modifications prior to arriving at wearables and equipment housings that were accepted widely by participants. Additional details of the modifications, samples of vests and area sampling equipment housings, feedback from participants as well as compliance information, are provided in Table S2 and Figure S2 of Supplementary Information (SI).

### Intervention adherence

Among pregnant women who were enrolled for before-and-after measurements, 100% adherence (i.e. use of LPG stove and fuel after installation as ascertained by direct household visits) was achieved throughout the 3 month intervention period. On the average, households received 2 cylinder refills in both the VP site and the NP site over the observation period. No stove or regulator repairs were requested by households. At the NP site, 10 households dismantled/removed their primary biomass stove out of their own volition. Qualitative feedback indicated overwhelming satisfaction with receiving the stove and free LPG refills (albeit only over a short period).

### PM_2.5_ exposures and area concentrations

**Fig. 2:**
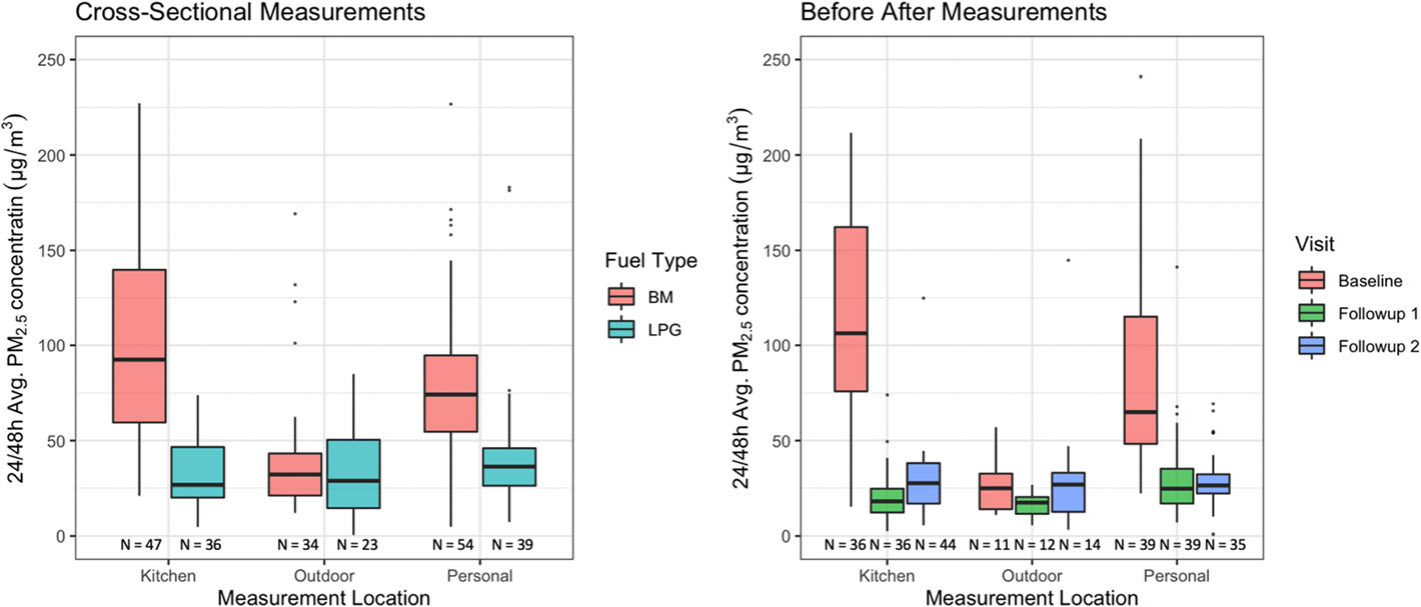
Distribution of 24/48 h kitchen area and near household (outdoor) PM_2.5_ concentrations and personal PM_2.5_ exposures for women and pregnant women. Values shown are pooled averages across measurements at both study sites, of concentrations/exposures measured across all instruments deployed for a specific type of measurement, during a specific 24/48 h period

We performed both 24 and 48-h personal exposure measurements. Sampling durations for kitchen and near-household (outdoor) measurements were matched to respective personal sampling durations. We pooled 24 or 48-h averages recorded in the cross-sectional arm and averages measured across all instruments in both cross-sectional and before-after arms based on observed correlations (*r* = 0.92 for 24 vs 48 h measures in the same households; *r* = 0.94 between SKC/Casella vs UPAS co-located measurements and *r* = 0.87 between SKC/Casella vs ECM co-located measurements). Scatter plots for observed correlations are shown in Supplementary Information (Figure S3). Distributions of the pooled measurement results are shown in Fig. 2 and Table 2.

**Table 2:** 24/48 h kitchen area and near household (outdoor) PM_2.5_ concentrations and personal exposures (μg/m^3^) for women and pregnant women

	Primary Fuel	Location	n	Mean	Median	Min	Max	SD	IQR
Cross-Sectional Measurements	Biomass (24 h)	Kitchen	46	217.7	132.1	20.9	1353	270.2	138.2
		Personal	41	100.9	73.4	4.6	967.4	143.7	36.1
		Outdoor	18	38	35.9	14.4	122.9	24.6	21.7
	Biomass (48 h)	Kitchen	18	227.1	151.2	27.8	1294.3	289.1	229.5
		Personal	15	100.2	80.1	21.3	280.1	75.1	70.1
		Outdoor	17	61.1	31.5	11.9	273.1	70.4	41.2
	LPG (24 h)	Kitchen	28	31.2	26.7	4.5	66	17.6	27.3
		Personal	26	33.6	35.8	7.1	53.5	11.2	16.1
		Outdoor	13	26.5	25.2	0.5	84.8	22.4	22.7
	LPG (48 h)	Kitchen	8	35.3	26.6	8.5	74	23.9	17.7
		Personal	13	63.6	48.3	12	182.9	56.1	48.3
		Outdoor	10	43.8	44.8	7.7	77.2	24.9	43.6
Before-After Measurements	Baseline Biomass (48 h)	Kitchen	36	277.9	160.3	15.2	1830.9	342.2	232.7
		Outdoor	11	26.2	24.9	10.8	57.1	15.4	18.8
		Personal	41	113.4	71.9	22.2	954.7	149.3	77.6
	Followup-1 LPG (48 h)	Kitchen	36	20.5	18	2.3	74.1	14.1	12.6
		Outdoor	12	16.7	17.35	5.4	26.7	6.5	8.9
		Personal	39	31.1	24.7	6.9	141.2	23.9	18.4
	Followup-2 LPG (48 h)	Kitchen	44	29.4	27.5	5.4	124.8	18.4	21.3
		Outdoor	14	31.7	26.8	3.1	144.8	35.1	20.6
		Personal	35	29.8	26.4	0.8	69.4	14.9	10.1

Note: Values shown are averages across measurements at both study sites for each type of measurement and across all instruments measuring concentrations/exposures during a specific 24/48 h period.

In the cross-sectional arm, for biomass and LPG-using homes, respectively, pooled median 24/48 h kitchen PM_2.5_ concentrations were 134 μg/m^3^ [IQR: 71–258] and 27 μg/m^3^ [IQR: 20–47], while median personal exposures for women were 75 μg/m^3^ [IQR: 55–104] and 36 μg/m^3^ [IQR: 26–46]. No significant differences were found between comparisons made using 24 vs. 48 h averages.

In the before-after arm the LPG fuel-and-stove intervention resulted in a substantial reduction in both kitchen concentrations and personal PM_2.5_ exposures. Median personal 48 h exposures for pregnant women were 72 μg/m^3^ [IQR: 49–127] at baseline while using biomass; 25 μg/m^3^ [IQR: 17–35] at the first month; and 26 μg/m^3^ [IQR: 22–32] at the second month after the LPG intervention. The LPG intervention thus resulted in a reduction of 93% in mean kitchen PM_2.5_ concentrations and 78% in mean personal PM_2.5_ exposures over the three-month intervention period.

Pooled median 24/48 h near-household (outdoor) concentrations were 31 μg/m^3^ [IQR: 21–43] and 21 μg/m^3^ [IQR: 11–35] in biomass- and LPG-using homes respectively. 48 h ambient concentrations were 23 μg/m^3^ [IQR: 15–27] with no significant differences across sites. Figure 2 shows the distribution of 24/48-h kitchen area, near outdoor concentrations, and personal PM_2.5_ exposures for women and pregnant women enrolled for cross-sectional and before-after measurements respectively.

The number of measurements performed using a particular instrument and sampling durations varied by site precluding a direct comparison of measurement results stratified by instrument, sampling duration, and site. Additional information regarding this is furnished in supplementary information (SI) (Figure S4 and Tables S3a, S3b, S3c). Despite, this limitation, the differences in concentrations/exposures between biomass and LPG using homes remained comparable across sites.

## Discussion

Trials of environmental health interventions are typically complex and are predicated on an expectation that interventions will reduce exposures and subsequently improve health. For studies of HAP, understanding existing sources of exposure and the potential for their reduction via a stove intervention is key to the success of the program. Often, these factors have not been well documented in previous literature. We provide here evidence of the ability of such an intervention to reduce exposures in rural Tamil Nadu, India. The exposure results reported here quantify the reduction in air pollution exposure that may be expected via an LPG stove intervention in Villuparum and Nagapattinam, Tamil Nadu, and strengthen the case for their selection for the HAPIN trial in India. As no prior HAP research has been conducted in these areas, the findings also provide site-specific baseline information relevant to global HAP reduction efforts and provide valuable information on LPG-related exposures in India.

### Exposure measurement feasibilities for main trial

We report results from 521 (210 personal, 216 kitchen area, 95 near household outdoor) 24/48 h PM_2._5 measurements in the districts of Villupuram and Nagapattinam. Further, we performed measurements under alternative scenarios of biomass and LPG use that could be expected in the sites: in our cross-sectional arm, our measurements represent routine fuel use in the study site; in our before-and-after arm, we mimic the HAPIN efficacy trial and performed measurements under ideal anticipated main trial conditions where households were provided with a licensed LPG connection, a high quality stove, free fuel, and behavioural reinforcements.

In addition to information on the exposure contrast (described below), this exercise confirmed many other exposure measurement-related feasibilities for the main trial. The selected study sites are located 250–450 km from SRIHER (the parent institution for study investigators in India) located in Chennai, India. We established temporary field offices to house staff and equipment, arranged for transportation logistics (for both staff and samples), coordinated exposure measurements alongside biomarker assessments, enabled maximal participant compliance through customization of clothing which held personal samplers, and augmented available analytical capacities in the SRIHER laboratory for filter weighing. Further, we made elaborate comparisons of device specific parameters related to charging and calibration as well as 24 vs. 48 h sampling durations that can be used alongside information from other HAPIN sites to refine main trial protocols. Finally, the HAPIN central core team built a large workforce of trained personnel at the India IRC for executing the complex monitoring protocols in preparation for the main trial.

### Exposure contrast

The use of LPG stoves and fuel by households at the proposed study sites in India was associated with significantly lower kitchen concentrations and personal exposures to PM_2.5_ when compared to use of biomass among women and pregnant women. Only a handful of studies that include information on HAP exposures among pregnant women [19, 39–42] or personal exposures associated with a LPG intervention [43] are currently included in the Global Database of HAP measurement studies. Our dataset thus adds important HAP related exposure information to the global database.

We observed sustained reductions (90 to 93%) in kitchen concentrations and (73 to 78%) in personal PM_2.5_ exposures for pregnant women over a 2 month follow up period (after an LPG stove/free fuel intervention) with post–intervention values consistently below the World Health Organization annual average Interim-1 PM_2.5_ Target (WHO-ITG1) of 35 μg/m^3^.

Recent studies involving clean fuel interventions among pregnant women report levels that are well above the WHO-ITG1 levels. Unmeasured traditional stove use and high levels of background ambient concentrations have been cited as possible reasons for post intervention kitchen concentrations around 76 μg/m^3^ in India [42] and personal exposures ranging from 61 to 118 μg/m^3^ in a recent RCT in Nigeria [19]. A study in Cameroon (LACE-1) that evaluated a national LPG rollout effort [43], observed low levels of personal exposures for women and children among primary LPG users (14.0 μg/m^3^ and 13 μg/m^3^), but also observed low levels amongst biomass users at baseline (46.0 μg/m^3^ and 27 μg/m^3^) and attribute this to short durations spent near the cook stove during the dry season (which was also the monitoring period).

Exposure contrasts for PM_2.5_ have seldom been described as part of pilot studies for HAP RCTs. The feasibility study for the RCT in Malawi was conducted using the “Chitetozo” improved biomass cook-stove. Personal CO was monitored on just 4 participants over two separate 24-h periods with no significant differences between baseline and follow up [24]. The investigators also note that monitoring was challenging and the additional cost of transport for deployment and retrieval of equipment could be overwhelming in the main study. The pilot study for the RCT in Nigeria used the” Stovetec” improved biomass cook-stove and reported a reduction in median kitchen PM_2.5_ concentrations from 1414 μg/m^3^ to 130 μg/m^3^, during the cooking period [44]. Perhaps at least partially as a result of these unimpressive pilot results, the stoves selected for the main trials in the Malawi and Nigeria RCTs were different from those tested in the pilot phase [18, 19], but initial feasibility results on exposures from these stoves were not presented separately.

Several recent RCTs have incorporated stove interventions using clean fuels (such as LPG and ethanol), but likewise few have presented exposure results. Among trials recently concluded in Ghana, Malawi, Nepal, Peru and Nigeria [18, 26–28], the Nigeria [19] and Malawi trials [45] have reported exposure results to date, demonstrating no significant difference between the control and treatment arms.

The exposure contrast results from the pilot phase of the HAPIN trial in India provide comprehensive and substantive evidence of the suitability of the candidate sites and proof of principle that large sustained reductions in personal exposure are feasible.

### Implications for anticipated health benefits

The exposure reductions achieved with the introduction of LPG among biomass users and the prevalent differences between existing LPG and biomass users suggest that use of LPG is likely to result in health benefits based on known exposure-response relationships for acute lower respiratory infections in infants, birth-weight, and blood pressure in HAP settings [16]. Further, using concentration-response relationships derived for pregnancy-period PM_2.5_ exposures and birthweight in a recent cohort study conducted by the same investigators in Tamil Nadu [40], we estimate a 82 g [95%CI: 62 to 103 g] gain in birth weight, to be associated with the exclusive use of LPG, among biomass users at the study sites in Tamil Nadu. These estimates are similar to gains of 86 g [95%CI, 56–117] in birth weight associated with LPG use, derived from meta-analyses (of 19 HAP observational studies based on categorical indicators of fuel use [46]); as well as gains of 88 g [95% CI, − 18 to 194] in birth weight resulting from the use of an ethanol stove/fuel in an RCT in Nigeria [19]. The observed consistency in these results provide further support for the estimated exposure contrasts as being favorable for the conduct of the HAPIN trial at the candidate sites in India.

### Study limitations

The investigators optimized available resources to generate the most relevant pieces of information for site selection for the HAPIN trial within a short time period of 9 months. However, this posed some limitations. We could not add a control arm for the before-after measurement group. This may have resulted in some residual confounding. Seasonal differences were minimal at the study sites over the period of the intervention, making it unlikely that associated household practices (such as boiling bath water and using the biomass cook-stove for heating) explain observed differences. The seasonal variations at the sites are also otherwise minimal with temperatures remaining in the hot temperate range throughout the year (https://mausam.imd.gov.in/).

Intervention adherence was complete and supported by regular observations and feedback at focused group discussions. However, simultaneous stove use monitoring would have supported this observation considerably. Further, we monitored LPG use over a several month period of time and may not have fully captured typical stove use patterns over longer times. While intervention use has been known to fluctuate over time [47], there is limited precedence for LPG refills being provided free of cost, along with a second cylinder to ensure continuity of supply [42]. The overwhelming positive feedback from participants about their willingness to use LPG exclusively in the study indicates that exclusive use is likely to be maintained under conditions of free supply (as is planned during the main trial).

The field staff made several observations that may have had a bearing on the observed exposure contrast (Table S3a,S3b, and S3c) including (i) shortened cooking durations and time spent near the stove on account of extreme hot weather that prevailed during much of the monitoring period; (ii) diminished compliance on account of the burden of excessive instrumentation (on account of co-locations of the ECM or UPAS devices with the SKC/Casella/Aircheck devices) in the cross-sectional phase of measurements; (iii) households choosing not to cook on some days, including on monitoring days (*n* = 3); and (iv) occasional use of the secondary biomass stove in LPG using households during the cross-sectional phase of measurements (*n* = 2). While these effects are most likely non-differential between LPG and biomass users (Cross-sectional arm) or between baseline and follow-up (before-after arm), recording these observations will allow us to address these more carefully in the analyses for the main trial.

We conducted a limited number of ambient measurements at the sites using real-time instrumentation. The measured concentrations remained low and stable throughout the monitored period. While initial site selection (that included field observations and surveys, described elsewhere) indicated an absence other major local sources, including brick kilns or hazardous industries, contributions from sources such as garbage or agricultural burning could be important and may not have been fully captured by the sparse density of ambient measurements. Further, contributions from second-hand smoke (from smokers in the household), smoke from neighbours, use of kerosene lamps as a secondary lighting source, and dust could attenuate differences in personal exposures [48, 49]. Fully addressing differential sources and/or exposure attribution was outside the scope of this pilot exercise, but will be addressed as part of the main trial.

Finally, we could not measure child exposures in the HAPIN pilot phase in India, as has been done at the HAPIN Guatemala site [50] on account of equipment [42] and expertise shortages. However, this will be addressed as part of HAPIN main trial activities.

## Conclusions

The HAPIN RCT is a very complex environmental health trial aimed at demonstrating the health impacts of an LPG stove and fuel intervention among four diverse biomass using communities in the countries of India, Guatemala, Rwanda and Peru. Results from the HAPIN trial can be very valuable for policy makers in the respective countries to draw implications of an LPG intervention for exposure reductions and health benefits for pregnant women, young children and older women among rural populations. Evidence from the study has the potential to considerably augment the global thrust on expanding clean household energy solutions.

Given the potential burden to participants, field staff, and others in the community, it was critical to establish feasibility among multiple axes at potential field sites in India and the other HAPIN IRCs. We find it critical that site selection processes be described comprehensively to afford insights into baseline conditions. Further, given the relative paucity of results from pilot studies for HAP RCTs in published literature, the methods and results add important information for the design of future research studies. Finally, this study adds valuable kitchen concentration and exposure data for LPG, which is generally lacking from the wider literature.

Recently published study protocols of the HAPIN main trial draw heavily on the results from the pilot phase across all countries [31–33]. The exposure monitoring results from pilot phase activities of the HAPIN trial provide evidence for suitability of the sites on the basis exposure contrast while also adding important information for trial feasibilities and the global pool of HAP exposure information in relation to clean cooking interventions. Results from the HAPIN main trial are expected to greatly augment available evidence for HAP interventions globally and we hope the results from the current study provide promising support for successful conduct of the trial at the selected sites in India.

## Supplementary Information

**Additional file 1.**

## Data Availability

The manuscript does not contain any individual data. All de-identified data used for analyses in this paper is available in readily accessible Microsoft Excel files that are stored in a secure server maintained by Emory University. Access can be provided upon request by the Data Management Core of the HAPIN trial upon receipt of email addressed to the corresponding author (kalpanasrmc@ehe.org.in).

## Abbreviations

BEC: Behavioral and Economics Core
BMI: Body Mass Index
CO: Carbon Monoxide
ECM: Enhanced Children’s MicroPEM
HAP: Household Air Pollution
HAPIN: Household Air Pollution Intervention Network
IQR: Interquartile Range
IRC: Intervention Research Center
LMICs: Low- and Middle-Income Countries
LPG: Liquefied Petroleum Gas
NP: Nagapattinam
RCTs: Randomized Controlled Trials
SI: Supplementary Information
SRIHER: Sri Ramachandra Institute for Higher Education and Research
UPAS: Ultrasonic Personal Air Sampler
VP: Villupuram
WHO: World Health Organization
