# Supplementary material for: Exposure contrasts associated with a liquefied petroleum gas (LPG) intervention at potential field sites for the multi-country household air pollution intervention network (HAPIN) trial in India: results from pilot phase activities in rural Tamil Nadu
Source: BMC Public Health. 2020 Nov 26;20:1799. doi: 10.1186/s12889-020-09865-1 (PMC7690197; doi:10.1186/s12889-020-09865-1)
Supplement: Supplementary file 1 — Additional file 1. [file 12889_2020_9865_MOESM1_ESM.docx]

**Supplementary Information**

**Table of Contents**

[**PM_2.5_ measurement protocols 1**](#_Toc42417116)

[Figure S1: Pictures of LPG regulator), two burner stainless steel LPG stove), hose (LPG pipe), LPG stove assembly with stand 2](#_Toc42417117)

[Figure S2: Pictures of wearables and equipment housing used across the VP and NP villages 3](#_Toc42417118)

[Table S1: Characteristics of participants and households enrolled for exposure measurements across study sites 3](#_Toc42417119)

[**Participant feedback on wearables, devices and equipment housings 4**](#_Toc42417120)

[Table S2: Compliance indicators for personal measurements 5](#_Toc42417121)

[Figure S3: Inter-monitor correlations for 24/48 hr kitchen concentrations and personal exposures for ECM vs SKC samplers (Right); UPAS vs SKC samplers (Center) and 24 vs.48-hr correlations for measurements using SKC samplers (Left). 6](#_Toc42417122)

[Figure S4: Distribution of 24/48 hr kitchen area and near household (outdoor) PM2.5 concentrations and personal PM2.5 exposures for women and pregnant women. 7](#_Toc42417123)

[Table S3a: 24/48 hr kitchen area and near household (outdoor) PM2.5 concentrations and personal PM2.5 exposures for women (cross-sectional phase) across sites. 8](#_Toc42417124)

[Table S3b: 24/48 hr kitchen area and near household (outdoor) PM2.5 concentrations and personal PM2.5 exposures [µg/m3] for pregnant women (before-after arm) 9](#_Toc42417125)

[Table S3c: Instrument wise averages 24/48 hr kitchen area and near household (outdoor) PM_2.5_ concentrations and personal PM_2.5_ exposures for women (cross-sectional phase) and pregnant women (before-after measurements) 10](#_Toc42417126)

[**Implications for on-going LPG initiatives in India 12**](#_Toc42417127)

[Figure S5: Potential effects of reduced median PM2.5 kitchen concentrations on birth weight (BW) in the cross-sectional (top) and before-after (bottom) phases of measurement 13](#_Toc42417128)

[Table S4: Results from applying median kitchen concentration estimates derived from cross sectional and before-after measurements in previously published exposure-response models for PM2.5 and birthweight in Tamil Nadu, India 13](#_Toc42417129)

[**References 14**](#_Toc42417130)

PM_2.5_ measurement protocols

Samples were collected at flow rates of 1.5 lpm on the Casella/Aircheck/SKC pumps and 1 lpm on the UPAS using 37mm Teflo filters (Pall Life Sciences, Port Washington, NY, USA) and 0.3 lpm on the ECMs using 15mm Teflo filters (PT15-AN-PF02, MTL LLC., Minneapolis, MN USA). Flow rates were calibrated (Gilian Gilibrator-2, Sensidyne, USA and TSI, Model- 4199, USA) prior to sampling and measured after completion of sampling. The ECM also collected nephelometric real-time data (not shown). Ambient samples were collected at a flow rate of 5 lpm on MiniVol^TM^ portable air sampler using 47 mm Teflon^TM^ filters (Pall Corporation) through a size selective PM_2.5_ impactor.

For personal exposure measurements, a customized vest was designed to carry multiple sampling devices (Figure S2). All participants were advised to wear the vest throughout the monitoring period, except during bathing and sleeping. During this period, participants were instructed to place the vest with the instruments less than 1 meter away from them. Special metal stands were designed to hold instruments for area measurements. Instruments were placed 1.5 meters above ground, 1 meter away from any doors and windows if possible, and in the kitchen, 1 meter away from the combustion zone of the primary cook-stove.

Filters were weighed in a temperature (20-23^o^C ±2^o^C) and humidity (30-40% ±5%) controlled environment at the SRIHER laboratory. Prior to weighing, the filters were equilibrated for 24 hours and then weighed on a microbalance (Sartorius, MSA6.6S000DF, Sartorius Lab Instruments GmbH & Co, Germany). Immediately before weighing, the filters were placed before a static neutralizer (STAT-FAN O-dc/YIBO1-ODR, Sartorius Lab Instruments GmbH & Co, Germany) for 10 seconds. Filters were weighed in duplicate and a third measurement was made if the filter mass of first two values varied by more than 5µg. Filters were transported at 4^o^C using dry ice and stored at -20^o^C prior to weighing. For each sample, PM_2.5_ mass was calculated by subtracting the median field blank mass from the sample mass. The final PM_2.5_ concentration was estimated by dividing the blank corrected filter masses by the volume of air sampled by the pump over the measurement period.


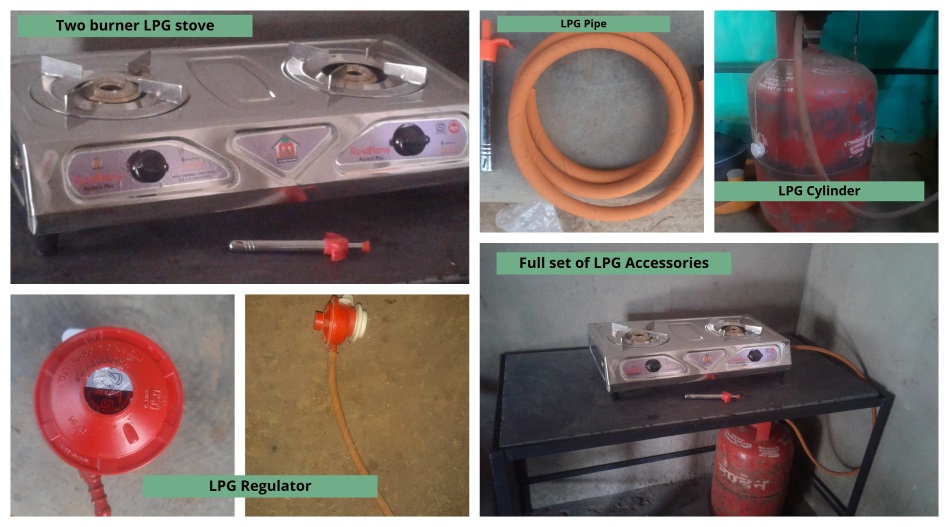


Figure S1: Pictures of LPG regulator), two burner stainless steel LPG stove), hose (LPG pipe), LPG stove assembly with stand

(Source: Gurusamy Thangavel, India site Co-PI for the HAPIN Trial; Reproduced with consent from participant to publish image)


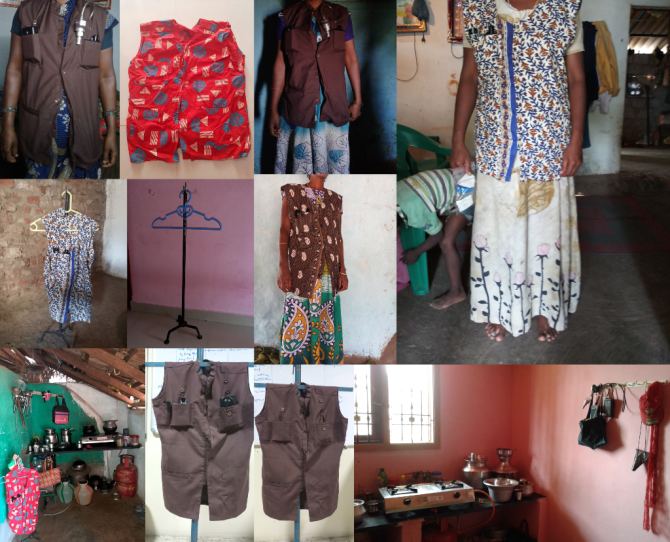


Figure S2: Pictures of wearables and equipment housing used across the VP and NP villages

(Source: Gurusamy Thangavel, India site Co-PI for the HAPIN Trial; Reproduced with consent from participant to publish image)

Table S1: Characteristics of participants and households enrolled for exposure measurements across study sites

| **Characteristics of women participants** | **Cross-Sectional Measurements** | | **Before After Measurements** | |
| --- | --- | --- | --- | --- |
|  | **VP** | **NP** | **VP** | **NP** |
| No of women/ pregnant women | 42 | 39 | 23 | 18 |
| Age in years, mean(SD)) | 36.5(9.7) | 38.3(11.2) | 23.5(2.5) | 23.7(3.2) |
| Number of school years( women), |  |  |  |  |
| mean(SD) | 1.3(2.8) | 3.6(3.5) | 5.2(5) | 10.6(3.5) |
| Primary cook,n(%) | 36(86) | 35(90) | 23(100) | 16(89) |
| **Household Characteristics** |  |  |  |  |
| **Kitchen characteristics** |  |  |  |  |
| Fully Enclosed(roof with 4 walls),n(%) | 42(100) | 38(97) | 18(78) | 15(83) |
| Kitchen size in m^2^, mean (SD) | 9.5(3.3) | 9.8(5.2) | 10.8(4.8) | 9.3(3) |
| Kitchen height in m, mean (SD) | 2.8(0.6) | 3.2(0.7) | 2.7(0.7) | 2.8(0.5) |
| **Kitchen Type, n(%)** |  |  |  |  |
| Separate building | 5(12) | 11(28) | 2(9) | 5(28) |
| Separate room attached to main house | 21(50) | 24(62) | 10(43) | 9(50) |
| Main living area in house | 16(38) | 4(10) | 8(35) | 2(11) |
| **Primary cooking fuel/stove, n(%)** |  |  |  |  |
| Biomass | 27(64) | 24(62) | N/A* | N/A* |
| LPG stove | 15(36) | 15(38) | N/A | N/A |
| **Number of Stoves used, n(%)** |  |  |  |  |
| One Stove | 17(40) | 23(59) | 13(57) | 16(89) |
| Two stoves | 19(45) | 12(31) | 9(39) | 2(11) |
| More than two stoves | 6(14) | 4(10) | 1(4) | 0 |
| Venting above stove | 27(64) | 24(62) | 26(63) | 27(64) |
| **Other HAP sources** |  |  |  |  |
| **Main Lighting Source .n(%)** |  |  |  |  |
| Kerosene lamp | 2(5) | 2(5) | 2(9) | 2(11) |
| Electricity | 40(95) | 37(95) | 21(91) | 16(89) |
| **Secondary Lighting Source .n(%)** |  |  |  |  |
| Kerosene lamp | 32(76) | 24(62) | 16(70) | 11(61) |
| Other | 1(2) | 1(3) | 1(4) | 1(6) |
| **Main Heating Source .n(%)** |  |  |  |  |
| No heating | 20(48) | 36(92) | 9(39) | 16(89) |
| Cooking fire | 22(52) | 3(8) | 14(61) | 2(11) |
| **Other sources. n(%)** |  |  |  |  |
| Garbage Burning | 4(10) | 13(33) | 4(17) | 7(39) |
| Use of mosquito coils | 12(29) | 15(38) | 2(9) | 5(28) |
| Smoke from neighbour's home | 19(45) | 11(28) | 9(39) | 3(17) |
| Tobacco smoking | 17(40) | 16(41) | 9(39) | 11(61) |
| Incense burning | 33(79) | 31(79) | 20(87) | 16(89) |

*Note: Primary cooking fuel/stove for before-after measurements is not provided as by only biomass users were enrolled at baseline*

Participant feedback on wearables, devices and equipment housings

Our team fabricated multiple vests (for personal monitoring) and metal stands (for area monitoring). All women reported wearing the vests for while at home and cooking. During the first two weeks of monitoring, the biggest concern with compliance expressed by participants was related to executing their outdoor chores such as fetching water, going to the market (for non-working women), and wearing the vest while working (especially for women engaged in agriculture related work, who feared the equipment may fall out). Participants initially reported a high degree of thermal discomfort with the chosen (synthetic washable fabric). Following this feedback, we designed cotton vests (with designs picked by participants) and added multiple pockets and fasteners to address concerns of equipment safety and participant comfort.

The initial designs for metal stands to hold equipment for area measurements were extremely inconvenient to transport to households in the field vehicle and were difficult to place within the narrow confines of the kitchen and living areas. Obstruction and the fear of toppling the metal stands were often cited as reasons for moving the stands away from the main living spaces. People were quite willing to let samplers hang on their walls and were willing to ensure security of the equipment while placing it outdoors. Following this feedback, we fabricated frames with hooks that could be hung over the wall or a beam; there was widespread acceptance for this method of area sampling. A small, free-standing structure with a coat hanger was provided to hold the vest (during sleeping and bathing). All the measures taken to improve participant comfort increased compliance both in terms of durations of vest wearing and of keeping equipment close by. Table S2 provides additional details of participant compliance.

At the end of each monitoring cycle, we recorded qualitative feedback on the level of comfort/discomfort experienced by participants during the course of 24 or 48-hr personal monitoring. Of the three types of samplers deployed for personal measurements, participants reported the greatest level of discomfort with the SKC /Casella gravimetric pumps with the weight of the device and cyclone position causing the biggest discomfort. ECMs and UPAS devices were reported to be equally comfortable, although the power banks used to support the UPAS devices for 48–hr measurements caused considerable discomfort.

Finally, research staff feedback also indicated that the calibration protocols provided by the manufacturer for the ECMs were lengthy (requiring 30-45 minutes per instrument). They identified the need for additional flow calibrators, laptops, and human resources for calibrating the large volume of samplers that would be needed in the main trial.

Table S2: Compliance indicators for personal measurements

|  |  |  |  |  |
| --- | --- | --- | --- | --- |
| Variables | Cross-Sectional Measurements | Before After Measurements | | |
|  |  | Baseline | Followup 1 | Followup 2 |
| **Women/Pregnant Women wearing the vest at the time of HH visit/sampler removal,**  **n(%)** |  |  |  |  |
| Yes | 26(32) | 33(80) | 33(82) | 27(68) |
| No | 29(36) | 8(20) | 7(18) | 13(32) |
| **If not, Was the equipment close to them? N(%)** |  |  |  |  |
| Yes | 16(55) | 6(75) | 4(57) | 8(62) |
| No | 13(45) | 2(25) | 3(43) | 5(38) |
| **Duration of Women/Pregnant women not wearing the vest during sampling period**  **Hrs, Mean(SD)** | 13.9(4.5) | 10.5(2.2) | 11.0(2.8) | 10.1(2.0) |


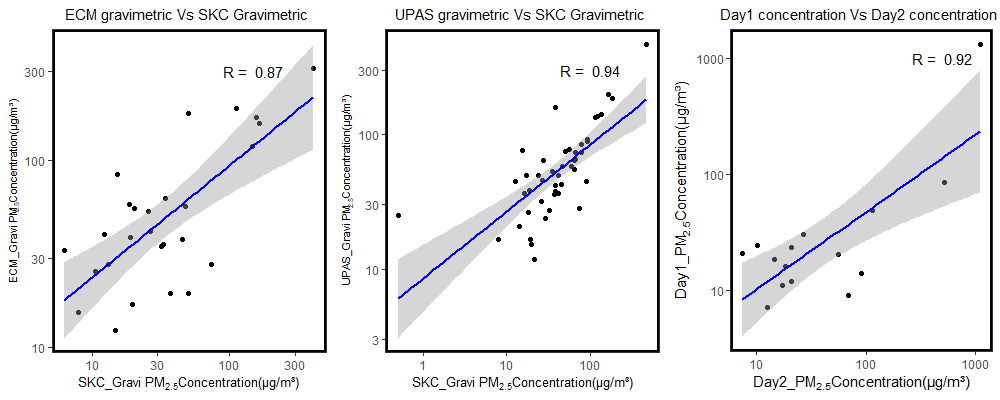


Figure S3: Inter-monitor correlations for 24/48 hr kitchen concentrations and personal exposures for ECM vs SKC samplers (Right); UPAS vs SKC samplers (Center) and 24 vs.48-hr correlations for measurements using SKC samplers (Left).

Note: ECM and SKC/Casella/AirCheck pumps were co-located in 14 households; UPAS and SKC/Casella/AirCheck pumps were co-located in 34 households; all instruments were co-located in 7 households


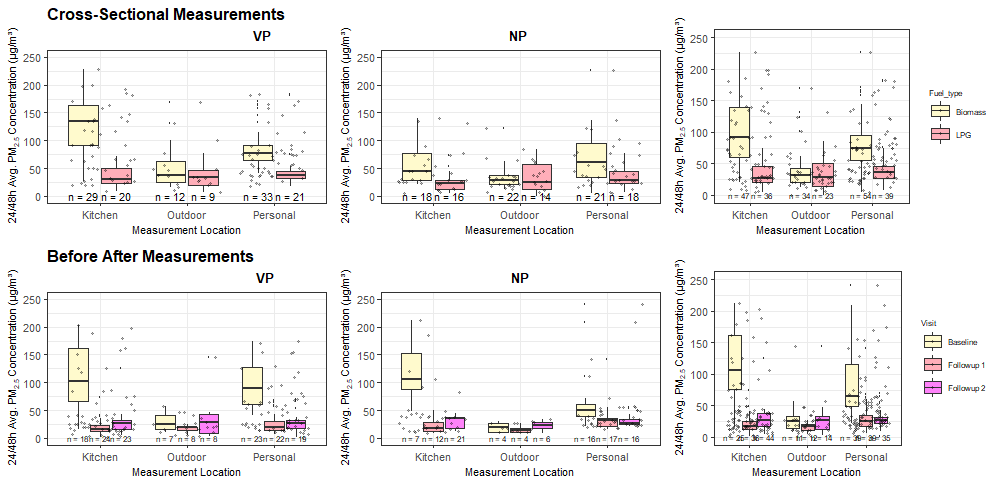


Figure S4: Distribution of 24/48 hr kitchen area and near household (outdoor) PM2.5 concentrations and personal PM2.5 exposures for women and pregnant women. Values shown are pooled averages of concentrations/exposures measured across all instruments deployed for a specific type of measurement, during a specific 24/48 hr period.

Table S3a: 24/48 hr kitchen area and near household (outdoor) PM2.5 concentrations and personal PM2.5 exposures for women (cross-sectional phase) across sites. Values shown are pooled averages of concentrations/exposures measured across all instruments deployed for a specific type of measurement, during a specific 24/48 hr period.

|  |  |  |  |  | PM_2.5_ concentrations/exposures [µg/m^3^] | | | | | | | |
| --- | --- | --- | --- | --- | --- | --- | --- | --- | --- | --- | --- | --- |
| **Study Arm** | **Site** | **Primary Fuel** | **Location** | **n** | **Mean** | **Median** | **Min** | **Max** | **SD** | **IQR** | **Q1** | **Q3** |
| Cross-Sectional Measurements | NP | Biomass  (24-hr) | Kitchen | 16 | 180.6 | 83.7 | 20.9 | 787.9 | 220.5 | 145.9 | 43.0 | 188.9 |
|  |  |  | Personal | 12 | 65 | 63.8 | 4.6 | 136.8 | 40.7 | 55.4 | 35.5 | 90.9 |
|  |  |  | Outdoor | 13 | 37.7 | 32 | 14.4 | 122.9 | 28.6 | 22.6 | 18.6 | 41.2 |
|  |  | Biomass  (48-hr) | Kitchen | 9 | 243.8 | 65.6 | 27.8 | 1294.3 | 412.4 | 232.3 | 30.0 | 262.3 |
|  |  |  | Personal | 9 | 77.8 | 61.7 | 21.3 | 226.8 | 66.5 | 73.0 | 28.5 | 101.5 |
|  |  |  | Outdoor | 9 | 29.8 | 27.4 | 11.9 | 62.5 | 14.5 | 12.6 | 21.3 | 33.9 |
|  |  | LPG  (24-hr) | Kitchen | 12 | 21.7 | 19.4 | 4.5 | 53.2 | 15.1 | 16.8 | 10.1 | 26.9 |
|  |  |  | Personal | 10 | 27.6 | 25.8 | 7.1 | 46.8 | 11.9 | 15.0 | 21.0 | 36.0 |
|  |  |  | Outdoor | 9 | 24.9 | 17.5 | 0.5 | 84.8 | 25.7 | 25.4 | 7.8 | 33.2 |
|  |  | LPG  (48-hr) | Kitchen | 4 | 37.5 | 27.2 | 21.7 | 74 | 24.6 | 18.3 | 23.3 | 41.5 |
|  |  |  | Personal | 8 | 42.4 | 43 | 12 | 76.4 | 20.3 | 26.0 | 28.4 | 54.4 |
|  |  |  | Outdoor | 5 | 48.9 | 64.1 | 20.3 | 67.9 | 23.5 | 39.5 | 26.3 | 65.8 |
|  | VP | Biomass  (24-hr) | Kitchen | 30 | 237.5 | 138.1 | 27.7 | 1353 | 295 | 126.6 | 91.7 | 218.3 |
|  |  |  | Personal | 29 | 115.7 | 76.1 | 40.9 | 967.4 | 167.6 | 31.4 | 59.6 | 91.0 |
|  |  |  | Outdoor | 5 | 38.8 | 38.1 | 23.3 | 48.9 | 10.5 | 12.5 | 35.6 | 48.1 |
|  |  | Biomass  (48-hr) | Kitchen | 9 | 210.5 | 198.3 | 115.9 | 322.6 | 83.1 | 169.7 | 138.9 | 308.6 |
|  |  |  | Personal | 6 | 133.9 | 98.9 | 74.1 | 280.1 | 80.3 | 76.8 | 80.6 | 157.4 |
|  |  |  | Outdoor | 8 | 96.3 | 66.7 | 15.9 | 273.1 | 91.8 | 116.0 | 25.2 | 141.2 |
|  |  | LPG  (24-hr) | Kitchen | 16 | 38.4 | 34.4 | 19.3 | 66 | 16.2 | 24.4 | 25.2 | 49.6 |
|  |  |  | Personal | 16 | 37.3 | 36.8 | 17.2 | 53.5 | 9.2 | 8.1 | 34.8 | 43.0 |
|  |  |  | Outdoor | 4 | 30 | 31.2 | 11.4 | 46.1 | 14.4 | 12.4 | 24.5 | 36.8 |
|  |  | LPG  (48-hr) | Kitchen | 4 | 33.1 | 26.5 | 8.5 | 71 | 26.7 | 19.9 | 19.8 | 39.7 |
|  |  |  | Personal | 5 | 97.4 | 74.8 | 21.6 | 182.9 | 80.1 | 154.8 | 26.5 | 181.3 |
|  |  |  | Outdoor | 5 | 38.7 | 34.7 | 7.7 | 77.2 | 27.9 | 35.8 | 19.0 | 54.8 |

Table S3b: 24/48 hr kitchen area and near household (outdoor) PM2.5 concentrations and personal PM2.5 exposures [µg/m3] for pregnant women (before-after arm) Values shown are pooled averages of concentrations/exposures measured across all instruments deployed for a specific type of measurement, during a specific 24/48 hr period.

|  | | | | | PM_2.5_ concentrations/exposures [µg/m^3^] | | | | | | | |
| --- | --- | --- | --- | --- | --- | --- | --- | --- | --- | --- | --- | --- |
| **Study Arm** | **Site** | **Primary Fuel** | **Location** | **N** | **Mean** | **Median** | **Min** | **Max** | **SD** | **IQR** | **Q1** | **Q3** |
| Baseline | NP | Biomass (48-hr) | Kitchen | 12 | 433.4 | 198.2 | 49.0 | 1830.9 | 510.0 | 517.4 | 102.8 | 620.1 |
|  |  |  | Personal | **17** | 122.4 | 50.6 | 22.2 | 954.7 | 223.4 | 30.6 | 41.3 | 71.9 |
|  |  |  | Outdoor | 4 | 19.7 | 19.1 | 10.8 | 29.7 | 9.9 | 16.0 | 11.4 | 27.4 |
|  | VP | Biomass (48-hr) | Kitchen | 24 | 200.2 | 155.0 | 15.2 | 833.3 | 186.7 | 143.1 | 82.1 | 225.2 |
|  |  |  | Personal | 24 | 107.0 | 91.8 | 40.9 | 329.5 | 63.1 | 72.5 | 62.5 | 135.1 |
|  |  |  | Outdoor | 7 | 29.9 | 24.9 | 10.9 | 57.1 | 17.3 | 24.5 | 16.9 | 41.4 |
| Followup-1 | NP | LPG  (48-hr) | Kitchen | 12 | 21.7 | 18.2 | 9.6 | 49.5 | 11.8 | 15.3 | 12.6 | 27.9 |
|  |  |  | Personal | 17 | 37.0 | 32.0 | 16.7 | 141.2 | 28.8 | 15.0 | 21.5 | 36.5 |
|  |  |  | Outdoor | 4 | 16.0 | 13.6 | 10.0 | 26.7 | 7.5 | 7.0 | 11.3 | 18.2 |
|  | VP | LPG  (48-hr) | Kitchen | 24 | 20.0 | 16.7 | 2.3 | 74.1 | 15.4 | 10.5 | 12.1 | 22.6 |
|  |  |  | Personal | 22 | 26.7 | 19.6 | 6.9 | 67.9 | 18.9 | 15.9 | 14.7 | 30.6 |
|  |  |  | Outdoor | 8 | 17.1 | 19.5 | 5.4 | 25.5 | 6.4 | 6.1 | 14.2 | 20.3 |
| Followup-2 | NP | LPG  (48-hr) | Kitchen | 21 | 29.8 | 36.6 | 12.3 | 44.6 | 11.7 | 19.7 | 18.6 | 38.3 |
|  |  |  | Personal | 16 | 31.4 | 26.6 | 20.8 | 54.9 | 10.8 | 9.2 | 24.7 | 33.9 |
|  |  |  | Outdoor | 6 | 22.5 | 22.4 | 9.7 | 34.5 | 9.0 | 10.5 | 17.6 | 28.1 |
|  | VP | LPG  (48-hr) | Kitchen | 23 | 29.1 | 26.2 | 5.4 | 124.8 | 23.2 | 14.9 | 16.6 | 31.5 |
|  |  |  | Personal | 19 | 28.5 | 26.2 | 0.8 | 69.4 | 17.8 | 13.7 | 17.8 | 31.5 |
|  |  |  | Outdoor | 8 | 38.6 | 27.9 | 3.1 | 144.8 | 45.8 | 33.3 | 9.6 | 42.8 |

Table S3c: Instrument wise averages 24/48 hr kitchen area and near household (outdoor) PM_2.5_ concentrations and personal PM_2.5_ exposures for women (cross-sectional phase) and pregnant women (before-after measurements)

|  |  |  |  |  | PM_2.5_ concentrations/exposures [µg/m^3^] | | | | | | | |
| --- | --- | --- | --- | --- | --- | --- | --- | --- | --- | --- | --- | --- |
| **Study Arm** | **Instrument** | **Primary Fuel** | **Location** | **n** | **Mean** | **Median** | **Min** | **Max** | **SD** | **IQR** | **Q1** | **Q3** |
| Cross-Sectional Measurements  Cross-Sectional Measurements | ECM | Biomass (24-hr) | Kitchen | 6 | 319.38 | 180.8 | 27.8 | 1094.1 | 390.9 | 151.7 | 131.7 | 283.4 |
|  |  |  | Personal | 5 | 98.46 | 113.4 | 4.6 | 158.2 | 59.3 | 50.9 | 82.6 | 133.5 |
|  |  |  | Outdoor | 1 | 58.6 | 58.6 | 58.6 | 58.6 | NA | 0.0 | 58.6 | 58.6 |
|  |  | Biomass (48-hr) | Kitchen | 9 | 190.69 | 198.3 | 27.8 | 370.8 | 131.1 | 243.0 | 65.6 | 308.6 |
|  |  |  | Personal | 11 | 117.93 | 101.5 | 28.5 | 280.1 | 79.1 | 79.0 | 67.9 | 146.9 |
|  |  |  | Outdoor | 10 | 86.51 | 49.6 | 20.6 | 273.1 | 83.8 | 100.9 | 23.4 | 124.2 |
|  |  | LPG  (24-hr) | Kitchen | 4 | 36.85 | 36.25 | 19.3 | 55.6 | 20.3 | 34.5 | 19.3 | 53.8 |
|  |  |  | Personal | 5 | 32.42 | 35.5 | 19.3 | 41.7 | 8.9 | 10.0 | 27.8 | 37.8 |
|  |  |  | Outdoor | 1 | 33.2 | 33.2 | 33.2 | 33.2 | NA | 0.0 | 33.2 | 33.2 |
|  |  | LPG  (48-hr) | Kitchen | 4 | 32.98 | 26.2 | 8.5 | 71 | 26.9 | 22.4 | 18.4 | 40.8 |
|  |  |  | Personal | 10 | 73.98 | 54.85 | 21.6 | 182.9 | 60.1 | 44.7 | 31.4 | 76.0 |
|  |  |  | Outdoor | 8 | 49.06 | 59.45 | 7.7 | 77.2 | 25.2 | 35.2 | 31.1 | 66.3 |
|  | SKC | Biomass (24-hr) | Kitchen | 28 | 180.2 | 123.05 | 20.9 | 787.9 | 185.6 | 79.8 | 83.5 | 163.2 |
|  |  |  | Personal | 20 | 117.22 | 73.15 | 16.8 | 967.4 | 202.7 | 36.1 | 53.6 | 89.7 |
|  |  |  | Outdoor | 16 | 36.7 | 33.8 | 14.4 | 122.9 | 25.6 | 21.5 | 20.4 | 41.9 |
|  |  | Biomass (48-hr) | Kitchen | 6 | 335.18 | 139.75 | 41.3 | 1294.3 | 480.1 | 198.8 | 84.0 | 282.9 |
|  |  |  | Personal | 1 | 21.3 | 21.3 | 21.3 | 21.3 | NA | 0.0 | 21.3 | 21.3 |
|  |  |  | Outdoor | 6 | 26.92 | 29.05 | 15.9 | 33.9 | 7.1 | 9.5 | 22.6 | 32.1 |
|  |  | LPG  (24-hr) | Kitchen | 19 | 26.74 | 25.5 | 4.5 | 66 | 16.4 | 19.8 | 13.5 | 33.3 |
|  |  |  | Personal | 14 | 30.07 | 29.9 | 7.1 | 45.2 | 11.2 | 15.1 | 23.5 | 38.6 |
|  |  |  | Outdoor | 11 | 25.96 | 17.5 | 0.5 | 84.8 | 24.4 | 26.3 | 9.4 | 35.7 |
|  |  | LPG  (48-hr) | Kitchen | 3 | 42.37 | 29.3 | 23.8 | 74 | 27.5 | 25.1 | 26.6 | 51.7 |
|  |  |  | Outdoor | 2 | 22.65 | 22.65 | 19 | 26.3 | 5.2 | 3.7 | 20.8 | 24.5 |
|  |  |  |  |  |  |  |  |  |  |  |  |  |
|  | UPAS | Biomass (24-hr) | Kitchen | 12 | 254.29 | 116.55 | 37.7 | 1353 | 366.1 | 160.5 | 74.1 | 234.6 |
|  |  |  | Personal | 16 | 81.24 | 73.15 | 33.8 | 163 | 37.0 | 32.6 | 56.8 | 89.4 |
|  |  |  | Outdoor | 1 | 38.4 | 38.4 | 38.4 | 38.4 | NA | 0.0 | 38.4 | 38.4 |
|  |  | Biomass (48-hr) | Kitchen | 3 | 120.37 | 134 | 28.4 | 198.7 | 86.0 | 85.2 | 81.2 | 166.4 |
|  |  |  | Personal | 3 | 61.7 | 80.1 | 22.9 | 82.1 | 33.6 | 29.6 | 51.5 | 81.1 |
|  |  |  | Outdoor | 1 | 11.9 | 11.9 | 11.9 | 11.9 | NA | 0.0 | 11.9 | 11.9 |
|  |  | LPG  (24-hr) | Kitchen | 5 | 43.92 | 45.8 | 26.9 | 65.7 | 15.4 | 17.8 | 31.7 | 49.5 |
|  |  |  | Personal | 7 | 41.37 | 42.4 | 25.2 | 53.5 | 9.6 | 11.9 | 36.2 | 48.1 |
|  |  |  | Outdoor | 1 | 25.2 | 25.2 | 25.2 | 25.2 | NA | 0.0 | 25.2 | 25.2 |
|  |  | LPG  (48-hr) | Kitchen | 1 | 23.6 | 23.6 | 23.6 | 23.6 | NA | 0.0 | 23.6 | 23.6 |
|  |  |  | Personal | 3 | 28.93 | 26.5 | 12 | 48.3 | 18.3 | 18.2 | 19.3 | 37.4 |
| Before After Measurements | ECM | Biomass (48-hr) | Kitchen | 20 | 285.95 | 181.85 | 49 | 833.3 | 250.8 | 298.5 | 110.5 | 409.0 |
|  |  |  | Personal | 26 | 142.45 | 91.8 | 22.5 | 954.7 | 181.2 | 103.5 | 51.5 | 154.9 |
|  |  |  | Outdoor | 4 | 34.23 | 31.05 | 17.7 | 57.1 | 16.9 | 16.5 | 24.4 | 40.9 |
|  |  | LPG  (48-hr) | Kitchen | 40 | 25.05 | 24.7 | 2.3 | 74.1 | 15.2 | 25.1 | 12.9 | 38.0 |
|  |  |  | Personal | 47 | 32.2 | 26.2 | 0.8 | 141.2 | 23.8 | 22.0 | 17.8 | 39.7 |
|  |  |  | Outdoor | 8 | 24.56 | 23.15 | 10 | 47.1 | 12.3 | 12.8 | 17.2 | 30.0 |
|  | SKC | Biomass (48-hr) | Kitchen | 3 | 34.3 | 37.8 | 15.2 | 49.9 | 17.6 | 17.4 | 26.5 | 43.9 |
|  |  |  | Outdoor | 6 | 22.53 | 18.25 | 10.8 | 47.3 | 14.6 | 17.4 | 11.1 | 28.5 |
|  |  | LPG  (48-hr) | Kitchen | 13 | 27.92 | 16.1 | 7.9 | 124.8 | 30.8 | 19.0 | 13.1 | 32.1 |
|  |  |  | Outdoor | 15 | 24 | 15.4 | 3.1 | 144.8 | 34.4 | 13.0 | 10.2 | 23.2 |
|  | UPAS | Biomass (48-hr) | Kitchen | 13 | 321.75 | 158.7 | 63.3 | 1830.9 | 474.2 | 203.2 | 92.2 | 295.4 |
|  |  |  | Personal | 15 | 63.03 | 56.5 | 22.2 | 118.5 | 28.4 | 28.8 | 47.5 | 76.3 |
|  |  |  | Outdoor | 1 | 16.1 | 16.1 | 16.1 | 16.1 | NA | 0.0 | 16.1 | 16.1 |
|  |  | LPG  (48-hr) | Kitchen | 27 | 24.82 | 21.9 | 9.8 | 44.6 | 10.0 | 14.4 | 17.4 | 31.8 |
|  |  |  | Personal | 27 | 27.64 | 25.2 | 14.7 | 54.9 | 10.4 | 11.9 | 20.8 | 32.6 |
|  |  |  | Outdoor | 3 | 29.3 | 26.4 | 20.1 | 41.4 | 10.9 | 10.7 | 23.3 | 33.9 |

Note: ECM and SKC/Casella/AirCheck pumps were co-located in 14 households; UPAS and SKC/Casella/AirCheck pumps were co-located in 34 households; all instruments were co-located in 7 households.

Implications for on-going LPG initiatives in India

Programmatic efforts to increase access to LPG in India have been operational for nearly two decades[1]. Over the last decade or so, the largest of these programs, Pradhan Mantri Ujjwala Yojana (PMUY) has provided LPG connections (i.e. a license to procure LPG through authorized LPG distributors) to nearly 80 million households, increasing LPG coverage in the country to nearly 95% of all households[2]. The biggest challenges to achieving health benefits from the PMUY program in its next phase is one of increasing LPG usage and eventually transitioning to exclusive use, as studies have shown that continued simultaneous use of solid fuels or stacking, as is common in India [3] may not produce health relevant exposure reductions[4]. Providing targeted support for vulnerable groups is now recognised as being critical for converting increased access to sustained usage[5]. Distributing free LPG during pregnancy has been shown to maximise usage throughout pregnancy and may encourage long-term behavioural change with minimal additional investments to increase reliability of supply and health messaging [6].

We estimated expected benefits for birth weight (one of the primary outcomes of the trial) associated with median differences in kitchen concentrations between biomass and LPG users, using concentration-response relationships derived in a recent cohort study conducted by the same investigators in Tamil Nadu [7]. Applying these differences on the C-R curves (Figure S5 and Table S4) we estimate a 82g [95%CI: 62 to 103g] gain in birthweight, associated with the exclusive use of LPG, among biomass users in Tamil Nadu. These estimates are similar to LPG use associated gains of 86g [95%CI: 56-117] estimated using results of meta-analyses [8] of 19 HAP studies based on categorical indicators of fuel use as well as gains from use of an ethanol stove/fuel of 88gms[95% CI: -18 to 194] estimated from an RCT in Nigeria [9].


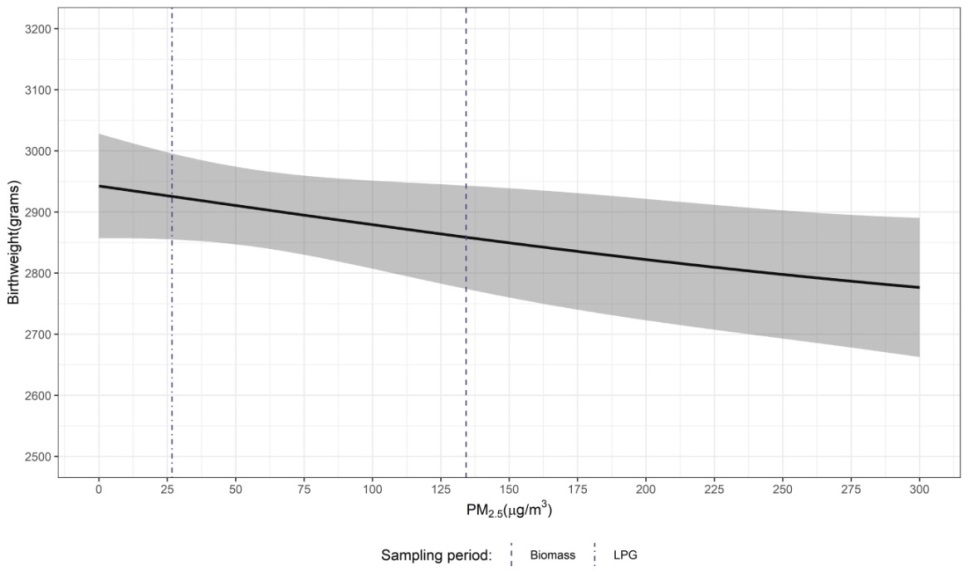


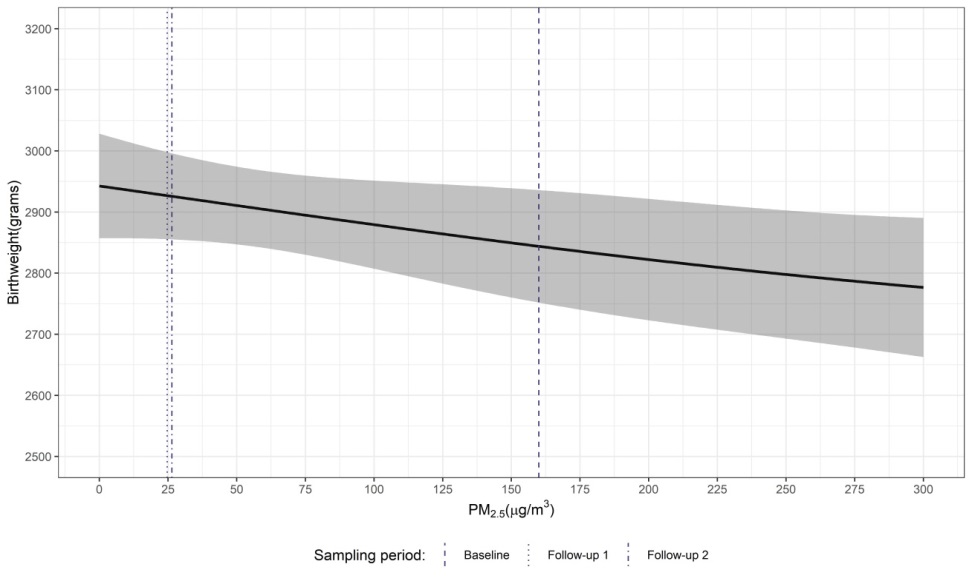


Figure S5: Potential effects of reduced median PM2.5 kitchen concentrations on birth weight (BW) in the cross-sectional (top) and before-after (bottom) phases of measurement (based on exposure-response relationships from Balakrishnan (2018) in Tamil Nadu, India). Grey shaded areas reflect 95% confidence intervals (model estimates are provided in Supplementary Table S4).

Table S4: Results from applying median kitchen concentration estimates derived from cross sectional and before-after measurements in previously published exposure-response models for PM2.5 and birthweight in Tamil Nadu, India

===========================================================================
 Cross-sectional measurements
===========================================================================
              PM_2.5_ Predicted Std.error   95%CI [low]95%CI[high]
 (µg/m^3^ (BW in gms)

Bio-mass     134.2 2858.77 43.10 2774.27 2943.26

LPG       26.7 2925.58 35.87 2855.26 2995.91

Diff (change) 107.5 -66.81   7.22  -80.98  -52.64

Before-After measurements
===========================================================================
              PM_2.5_    Predicted Std.error   95%CI [low]95%CI[high]
 (µg/m^3^ (BW in gms)

Baseline 160.0 2843.92   46.97 2751.85 2935.99

Follow Up      24.7 2926.86 36.34 2855.63 2998.09

Diff(change) 135.3 -82.94 10.63 -103.78 -62.10
===========================================================================Note: Model specifications are provided in Balakrishnan et al 2018.

The results from this interpolation provides important suggestive evidence to support such targeted initiatives for pregnant women, whereby it may be possible to scale sustained use of LPG to achieve potential health benefits. There is good precedence for success with pregnancy period interventions in India through the Janani Sishu Suraksha Karyakram (JSSK), that provides conditional financial support to poor pregnant women to adopt health behaviors during pregnancy (<https://nhm.gov.in/WriteReadData/l892s/97827133331523438951.pdf>. Last Accessed 06/-7/2020)

Evidence from this pilot study can support the initiation for a parallel effort for using LPG during pregnancy. The cost of providing refills throughout pregnancy are expected to range from ~$25 - $45 and will roughly increase the existing JSSK allocation per woman by 9-17%, adding feasibility for such a proposition while also providing substantial potential health benefits to unborn children, pregnant mothers, and other households.

References

1. Smith KR. Chapter 5 The Indian LPG Programmes: Globally Pioneering Initiatives. In: Bibek Debroy & Ashok Malik, eds, India@70; Modi@3.5. New Delhi: Wisdom Tree; 2017. p. 211pp.

2. Mani S, Jain A, Tripathi S, Gould CF. The drivers of sustained use of liquified petroleum gas in India. Nature Energy. 2020;:1–8.

3. Patnaik S, Saurabh T, Abhishek J. Roadmap for Access to Clean Cooking Energy in India. New Delhi: Council on Energy, Environment and Water.; 2019.

4. Johnson MA, Chiang RA. Quantitative Guidance for Stove Usage and Performance to Achieve Health and Environmental Targets. Environ Health Perspect. 2015;123:820–6.

5. Santosh H, Smith KR. Ujjwala 2.0 From Access to Sustained Usage. Policy Brief. New Delhi: Collaborative Clean Air Policy Centre; 2019.

6. Pillarisetti A, Ghorpade M, Madhav S, Dhongade A, Roy S, Balakrishnan K, et al. Promoting LPG usage during pregnancy: A pilot study in rural Maharashtra, India. Environment International. 2019;127:540–9.

7. Balakrishnan K, Ghosh S, Thangavel G, Sambandam S, Mukhopadhyay K, Puttaswamy N, et al. Exposures to fine particulate matter (PM2.5) and birthweight in a rural-urban, mother-child cohort in Tamil Nadu, India. Environmental Research. 2018;161:524–31.

8. Amegah AK, Quansah R, Jaakkola JJK. Household Air Pollution from Solid Fuel Use and Risk of Adverse Pregnancy Outcomes: A Systematic Review and Meta-Analysis of the Empirical Evidence. PLoS One. 2014;9. doi:10.1371/journal.pone.0113920.

9. Alexander DA, Northcross A, Karrison T, Morhasson-Bello O, Wilson N, Atalabi OM, et al. Pregnancy outcomes and ethanol cook stove intervention: A randomized-controlled trial in Ibadan, Nigeria. Environment International. 2018;111:152–63.
